# Is moderate-intensity interval training more tolerable than high-intensity interval training in adults with obesity?

**DOI:** 10.5114/biolsport.2023.123323

**Published:** 2023-05-15

**Authors:** Rami Maaloul, Imen Ben Dhia, Houssem Marzougui, Mouna Turki, Faten Hadj Kacem, Rihab Makhlouf, Mohamed Ben Amar, Choumous Kallel, Tarak Driss, Mohamed Habib Elleuch, Fatma Ayadi, Sameh Ghroubi, Omar Hammouda

**Affiliations:** 1 Research Laboratory, Molecular Bases of Human Pathology, LR19ES13, Faculty of Medicine, University of Sfax, Tunisia; 2 High Institute of Sport and Physical Education of Sfax, University of Sfax, Tunisia; 3 Research Laboratory: Evaluation and Management of Musculoskeletal System Pathologies, LR20ES09, Faculty of Medicine, University of Sfax, Sfax, Tunisia; 4 Endocrinology Department, Hedi Chaker University Hospital, Sfax, Tunisia; 5 Department of General and Digestive Surgery, Faculty of Medicine, University of Sfax, Habib Bourguiba Hospital, Sfax, Tunisia; 6 Department of hematology, Habib Bourguiba Hospital, Sfax, Tunisia; 7 Interdisciplinary Laboratory in Neurosciences, Physiology and Psychology: Physical Activity, Health and Learning (LINP2), UFR STAPS, UPL, Paris Nanterre University, Nanterre, France

**Keywords:** Interval exercise, Muscular damage, Perceived exertion, Inflammation, Obesity

## Abstract

Interval training (IT) has been shown to be a time-effective alternative to traditional training programmes in the management of obesity. Nevertheless, studies comparing the effects of different IT intensities on inflammation, muscle and liver damage, and perceptual responses in people with obesity are relatively scarce. This study aimed to compare the acute effects of two different IT protocols matched by the mean load and duration on biochemical and perceptual responses in sedentary adults with obesity. Twenty-two volunteers (age = 33.40 ± 10.01 years, BMI = 38.29 ± 7.09 kg/m²) were randomized to perform two conditions: moderate-intensity IT (MIIT) 5 × 3 min (70% of peak power output (PPO))/2 min (45%PPO) and high-intensity IT (HIIT) 8 × 1 min (90%PPO)/2 min (45%PPO). Blood samples were drawn before and after exercise for biochemical and haematological measurements. Rating of perceived exertion (RPE) was assessed during and after exercise. Perceptual pain was evaluated before, throughout and after exercise. C-reactive protein, white blood cells and neutrophils increased only after HIIT (p < 0.001, for all). Aspartate aminotransferase, alanine aminotransferase, creatine kinase and lactate dehydrogenase increased in both HIIT and MIIT (p < 0.001, for all), without any difference between sessions. HIIT induced a greater increase of blood lactate compared to MIIT (p < 0.05). Pain and RPE scores were higher during HIIT vs. MIIT (p < 0.001 and p < 0.01, respectively). MIIT induced fewer immune system perturbations and less muscle pain and was perceived as more tolerable compared to HIIT session. Therefore, MIIT could be used as a first step to promote body adaptations before starting a HIIT programme in sedentary people with obesity.

## INTRODUCTION

Excess body weight increases the incidence of morbidity and mortality from a myriad of life-threatening diseases [1]. A major link between these various disorders and obesity is a state of chronic low-grade inflammation characterized by infiltration and activation of several pro-inflammatory biomarkers (e.g., interleukin 6 (IL-6), tumour necrosis factor-alpha (TNF-α) and C-reactive protein (CRP)) [1].

Regular exercise can help to lose weight and to attenuate obesity-related comorbidities [2]. Despite the beneficial effects of moderate-intensity continuous exercise (MICT), the majority of adults with obesity fail to meet these recommendations due to various barriers such as physical limitations, and lack of time and motivation [3]. There is growing evidence encouraging the application of interval training (IT) as a time-efficient alternative to MICT for health improvement in the general population [4]. IT consists of intermittent periods of relatively intense effort interrupted with periods of active or passive recovery [4]. Nevertheless, when aiming to compare the effectiveness of IT in the management of excess body weight and its related health conditions, it has been usual to compare supra-maximal or near-maximal intensities with MICT [5], which are different not only in their intensities but also in the type of stimulus: intermittent versus continuous. Few studies have compared the chronic effects of different IT modes in the management of obesity. In this context, Naves et al. [6] reported that high-intensity IT (HIIT) and sprint IT improved anthropometric and cardiorespiratory fitness even without changes in dietary intake, yet only sprint IT reduced body weight in healthy young women. Other studies have investigated the efficacy of IT in people with obesity by comparing HIIT to moderate-intensity IT (MIIT). Alkahtani et al. [7] demonstrated a similar increase in fat oxidation and decrease in blood lactate (BLa) after both HIIT and MIIT, yet rating of perceived exertion (RPE) decreased to a greater extent following HIIT compared to MIIT. Racil et al. [8] indicated superior beneficial effects of HIIT versus MIIT on the levels of insulin, total cholesterol, and low-density lipoprotein cholesterol, without differences between the HIIT and MIIT groups for high-density lipoprotein cholesterol, insulin resistance and adiponectin. However, studies examining acute biological, metabolic and perceptual responses to different intensities of interval exercise in sedentary adults with high body mass index (BMI) are rare. To the best of the authors’ knowledge, only the study of Dorneles et al. [9] has compared the acute effects of HIIT and MIIT on the inflammatory response and muscle damage in males with overweight-obesity (BMI = 31.99 ± 3.93 kg/m^2^). The results showed a similar increase of white blood cells (WBC) and creatine kinase (CK) after HIIT versus MIIT, while only HIIT decreased the pro-inflammatory cytokine (i.e. IL-8) level and increased anti-inflammatory mediators (i.e. IL-6 and IL-10), indicating that HIIT was well tolerated by adults with overweight and obesity grade 1. Hence, the question that arises about the tolerance of HIIT in inactive adults having a high BMI is still unanswered. In this context, Kim et al. [10] reported a remarkable increase in muscle soreness and CK levels after a repetitive eccentric exercise in the high BMI group compared to the normal BMI group, indicating that BMI is one of the potential factors affecting muscle damage after intense exercise. Thus, this study aimed to compare the acute effects of HIIT versus MIIT on inflammation, muscle and liver damage, BLa and perceptual responses in sedentary adults with high BMI. We hypothesized that acute changes in markers of inflammation, cellular damage, BLa, perceived pain and RPE would be greater after HIIT compared to MIIT and that MIIT would be more tolerable in sedentary adults with high BMI.

## MATERIALS AND METHODS

### Participants

Twenty-two sedentary adults with obesity (8 men and 14 women) provided their written informed consent and completed the trial. Participants’ characteristics are presented in Table 1. Participants suffering from diabetes, autoimmune diseases, infections, cardiovascular diseases, musculoskeletal impairments, or any comorbidities limiting exercise were excluded. Participants were not engaged in any physical training programmes for the last six months. All female participants were eumenorrheic with menses length between 4 and 6 days and they had not been using hormonal contraceptives during the past six months. They also cautioned against taking any supplements or drugs that could affect the immune system throughout the study. This study was approved by the local Ethics Committee for the Protection of Persons and was conducted in compliance with the ethical guidelines of the Declaration of Helsinki for human experimentation.

**TABLE 1:** Participants’ characteristics.

Characteristic	Mean ± SD
**Age (years)**	33.40 ± 10.01
**Height (cm)**	168.95 ± 8.03
**Weight (kg)**	108.30 ± 24.02
**Body mass index (kg/m²)**	38.29 ± 7.09
**Body fat (%)**	42.01 ± 10.71
**Body fat (kg)**	46.79 ± 20.43
**Fat-free mass (kg)**	61.21 ± 10.05
**Basal metabolism (kcal)**	1909.45 ± 292.36
**Waist circumference (cm)**	111.88 ± 16.59
**Hip circumference (cm)**	128.54 ± 17.75
**Waist-to-hip ratio**	0.87 ± 0.89
**Peak power output (W)**	138.41 ± 42.91
**VO_2_ max (L · min^−1^)**	2.88 ± 1.09
**VO_2_ max (mL · kg^-1^ · min^−1^)**	26.58 ± 2.11
**Resting Heart rate (bpm)**	86.12 ± 9.07
**Diastolic blood pressure (mmHg)**	80.55 ± 11.47
**Systolic blood pressure (mmHg)**	120.41 ± 12.27

### Study design

Participants attended the rehabilitation unit on five occasions, where all assessments and exercise sessions took place. All testing sessions were performed between 7:00 and 10:00 a.m. after an overnight fast. Participants were instructed to abstain from any strenuous exercise for 72 h before all testing sessions and to replicate their food intake as closely as possible on the day before the exercise session. At the first visit, anthropometric measurements were assessed and the maximal graded exercise test was done. During the second and the third visit, two familiarization sessions were performed to familiarize the participants with HIIT and MIIT exercises. During the fourth and fifth visits, patients were randomly allocated to perform a HIIT or MIIT session. Free online software was used to randomize and counterbalance the order of the testing sessions. A seven-day washout period was applied between MIIT and HIIT sessions. The maximal graded exercise test, HIIT and MIIT were all performed using an electromagnetic ergo-cycle (Ergoselect 100, Ergoline, Germany).

### Maximal graded exercise test

The test started at 30 W with a progressive increase of 15 W/min [11]. Participants continued cycling until volitional exhaustion and/or attaining at least four of the following criteria: (a) RPE > 9; (b) heart rate > 90% of maximal heart rate; (c) respiratory exchange ratio ≥ 1.15; (d) inability to maintain a cycling cadence between 60 and 70 rpm; (e) VO_2_ steady state [12]. After reaching their maximum, a 3-min cool-down period at 0 W was applied. Blood pressure was measured every 2 min with a manual sphygmomanometer. Heart electrographic activity was monitored continuously using a 12-lead electrocardiogram. RPE was assessed at every stage with the 0–10 Borg scale [13]. Gas exchange was measured throughout the test using a gas analyser (Metasys TR-B, Brainware, France). The VO_2_ max was determined as the highest attained VO_2_ over the last 15 s of the test. The power of the last completed stage was considered as the peak power output (PPO).

### IT protocols

In both sessions, a 3-minute warm-up and cool-down of unloaded cycling were performed. The HIIT protocol consisted of 8 bouts of 1 min (90%PPO) alternated with 2 min of active recovery (45%PPO). The MIIT session involved 5 bouts of 3 min (70%PPO) interspersed with 2 min of active recovery (45%PPO). For the same participant, HIIT and MIIT protocols were matched by the mean load (P _mean_) and duration (≈24 min). P _mean_ was calculated using the formula of Tschakert and Hofmann [14]:

P_mean_ = (P_peak_ × T_peak_ + P_recovery_ × T_recovery_)/ (T_peak_ + T_recovery_)

where P_peak_, T_peak_, P_recovery_ and T_recovery_ are the peak workload intensity, the peak workload duration, the recovery load and the recovery duration, respectively (Table 2).

**TABLE 2:** Characteristics of high-intensity interval training (HIIT) versus moderate-intensity continuous exercise (MIIT).

	HIIT	MIIT
P _peak_ (%PPO)	90	70
T _peak_ (min)	1	3
P _recovery_ (%PPO)	45	45
T _recovery_ (min)	2	2
Sets	8	5
Total duration (min)	24	24
Females’ P _mean_ (W)	70.05	70.04
Males’ P _mean_ (W)	105.83	105.85
Females and males’ P _mean_ (W)	83.06	83.06

**Abbreviations:** HIIT, high intensity interval training; MIIT, moderate intensity interval training; PPO, peak power output; P _peak,_ peak workload intensity; T_peak_, peak workload duration; P _recovery,_ recovery load; T _recovery_, recovery duration; P _mean_, mean load.

### Anthropometric measurements and body composition

Height was measured to the nearest 0.5 cm using a wall-mounted stadiometer. A non-elastic tape was used to measure waist circumference (WC) and hip circumference (HC) according to the World Health Organization guidelines [15]. Waist-to-hip ratio was defined as WC divided by HC. Weight and body composition were measured using a bioelectrical impedance scale (Tanita BC-418, Tokyo, Japan).

### Blood sampling and analysis

Blood samples (8 ml) were drawn pre-exercise and 3–5 min after exercise cessation from the forearm vein in a sitting position. These samples were divided into three distinct blood collection tubes. The first EDTA tube was used to determine WBC, neutrophils, lymphocytes and monocytes using a multichannel automated blood cell analyser: Beckman Coulter Gen system-2 (Coulter T540). The second lithium heparin tube was used for aspartate aminotransferase (AST), alanine aminotransferase (ALT), CK, lactate dehydrogenase (LDH), and CRP analysis. The third sodium fluoride/potassium oxalate tube was used to determine BLa concentrations. Biochemical measurements were done through the Roche Cobas 6000 (c501) analyser. All specimens were placed in an ice bath to be centrifugated at 2500 r/min (× g) at 4°C for 10 min. Aliquots of the resulting plasma were stored at -80°C until analysed. To eliminate inter-assay variance, all samples were analysed in the same assay run. All assays were carried out in duplicate in the same laboratory with simultaneous use of a control serum from Randox. Changes in plasma volume induced by exercise were corrected using the formula of Dill and Costill [16]. All methods used in haematological [17] and biochemical analysis are listed in Table 3.

**TABLE 3:** Different used methods in blood analysis.

Parameter	Abbreviation	Method
**C-reactive protein**	CRP	Immunoturbidimetric (intra-assay CV = 1.16%)
**White blood cells count**	WBC	Standard laboratory method of Dacie and Lewis [17]
**Aspartate aminotransferase**	AST	340 nm kinetics (intra-assay CV = 1.1%)
**Alanine aminotransferase**	ALT	340 nm kinetics (intra-assay CV = 1.15%)
**Creatine Kinase**	CK	340 nm kinetics (intra-assay CV = 1.3%)
**Lactate dehydrogenase**	LDH	340 nm kinetics (intra-assay CV = 0.2%)
**Blood lactate**	BLa	Lactate oxidase peroxidase method

**Abbreviation:** CV, coefficient of variation.

### Perceptual records

**FIG. 1:**
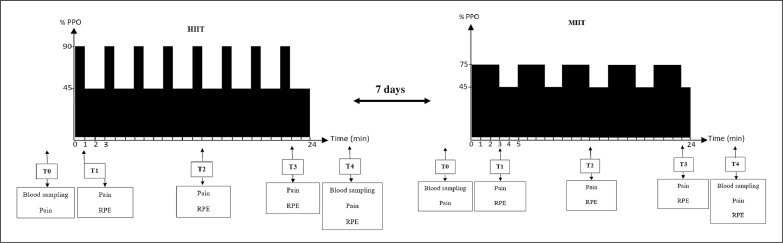
Simplified study design. HIIT, high-intensity interval training; MIIT, moderate-intensity interval training, T0, pre-exercise; T1, after the first bout; T2, at the middle of the session; T3, after the last bout; T4, 3 min post-exercise; RPE, rating of perceived exertion; PPO, peak power output.

Perceptual pain was evaluated by a 10 cm visual analogue scale with 0 = “no pain” and 10 = ” “maximum pain” [18]. RPE was assessed by the 0–10 Borg scale with 0 = “extremely easy” and 10 = “extremely hard” [13]. Measurements were done before exercise (T0), immediately after the first bout (T1), in the middle of the session (T2), the last bout (T3) and 3 min after exercise (T4) (Figure 1).

### Statistical analysis

Data are presented as mean ± standard error of the mean (SEM) in figures and as mean ± standard deviation (SD) in tables and analysed using IBM SPSS version 23.0 Software (SPSS Inc., Chicago, IL, USA). The normality of data was checked through the Shapiro-Wilk test. When the data were normally distributed, a two-way ANOVA with repeated measures [intensity (HIIT × MIIT) and time (pre-exercise × post-exercise)] was applied. If significant main effects were detected, Bonferroni post-hoc tests were performed. When the Shapiro-Wilk test was significant (p < 0.05), the Friedman test was applied. If appropriate, pairwise comparisons were performed using the Wilcoxon test. Effect sizes were calculated through partial eta-squared (ηp^2^) for the two-way repeated-measures ANOVA and by Kendall’s coefficient of concordance (Kendall’s W) when the data were skewed. The level of statistical significance was set at p < 0.05.

## RESULTS

**FIG. 2:**
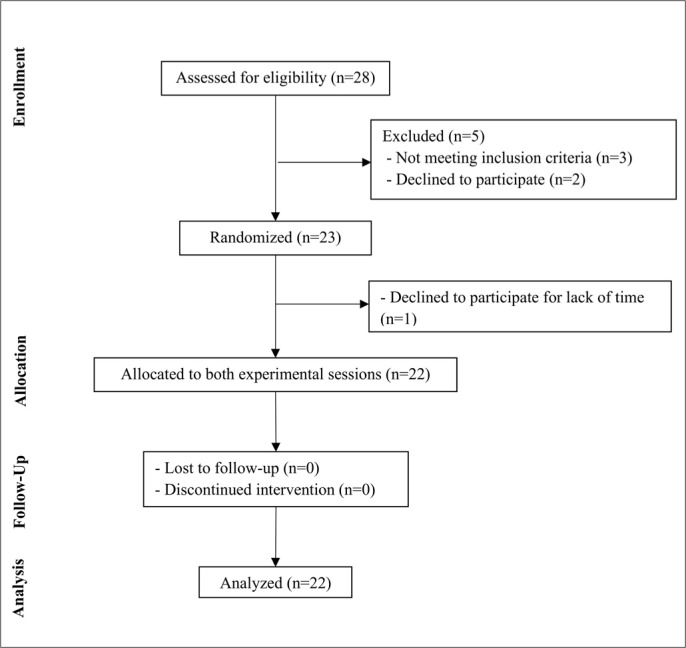
Flowchart of study participants.

Twenty-eight adults with obesity volunteered to participate and they were screened for eligibility. Twenty-three participants met all the eligibility criteria and were randomized to perform two different interval exercises, yet twenty-two completed the trial. Figure 2 summarizes the process of selection of volunteers.

### Acute effects of HIIT and MIIT on inflammatory and haematological biomarkers

The Friedman test revealed a significant effect (test = 21.54, p < 0.001, Kendall’s W = 0.33) for CRP levels. Pairwise comparison showed an increase in CRP levels only after HIIT (+10.78%, p < 0.001) with a slight, but not significant difference between exercise protocols (p = 0.07) (Table 4).

**TABLE 4:** Haematological and biochemical changes in response to acute high-intensity interval training (HIIT) versus moderate-intensity continuous exercise (MIIT).

	HIIT			MIIT		
Variables	Pre	Post	% change	Pre	Post	% change
**CRP (mg · L^-1^)**	4.36 ± 2.44	4.83 ± 2.48***	10.78	4.45 ± 2.42	4.54 ± 2.32	2.02
**WBC (10^3^/*μ*l)**	7.83 ± 2.37	8.96 ± 2.42***	14.43	7.74 ± 2.65	7.97 ± 2.43^###^	2.97
**Neutrophils (10^3^/*μ*l)**	4.55 ± 1.73	5.35 ± 1.89***	17.58	4.48 ± 1.82	4.71 ± 1.65^###^	5.13
**Lymphocytes (10^3^/*μ*l)**	2.41 ± 0.66	2.58 ± 0.75	7.05	2.44 ± 0.77	2.45 ± 0.76	0.41
**Monocytes (10^3^/*μ*l)**	0.55 ± 0.18	0.57 ± 0.16	3.64	0.54 ± 0.22	0.54 ± 0.18	0.00
**AST (U/l)**	17.05 ± 3.29	18.87 ± 3.47***	10.67	17.14 ± 2.72	18.47 ± 2.86***	7.76
**ALT (U/l)**	20.46 ± 7.01	21.77 ± 7.28***	6.40	19.99 ± 5.63	20.95 ± 6.53**	4.80
**CK (U/l)**	128.45 ± 44.08	141.45 ± 52.69***	10.12	118.82 ± 59.60	129 ± 68.86***	8.57
**LDH (U/l)**	186.59 ± 33.40	201.23 ± 33.98***	7.85	181 ± 34.97	198.5 ± 37.28***	9.67
**BLa (mmol · L^-1^)**	1.13 ± 0.37	4.30 ± 2.71***	280.53	1.12 ± 0.38	3.46 ± 1.82 ^***, #^	208.93

Data are presented as mean ± SD. **Abbreviations:** HIIT, high-intensity interval training; MIIT, moderate-intensity interval training; WBC, white blood cells; CRP, c-reactive protein; AST, aspartate aminotransferase; ALT, alanine aminotransferase; CK, creatine kinase; LDH, lactate dehydrogenase; BLa, blood lactate.

**, *** : significant difference between pre-post exercise (p<0.01, p<0.001, respectively).

#, ### : significant difference between HIIT-MIIT (p<0.05 and p<0.001, respectively).

Statistical analysis of WBC showed a significant effect of time (F_(1, 21)_ = 21.25, p < 0.001, ƞp² = 0.5) and intensity × time interaction (F_(1, 21)_ = 14.23, p < 0.01, ƞp² = 0.4). WBC increased significantly only after HIIT (+14.43%, p < 0.001) with a significant difference between exercise sessions (p < 0.001). Similarly, neutrophils demonstrated a significant effect of time (F_(1, 21)_ = 19.29, p < 0.001, ƞp² = 0.48) and intensity × time interaction (F_(1, 21)_ = 12.59, p < 0.01, ƞp² = 0.38). Neutrophils increased only following HIIT (+17.58%, p < 0.001) with a significant difference between HIIT and MIIT (p < 0.001). No significant effects were observed for monocytes and lymphocytes.

### Acute effects of HIIT and MIIT on muscle and hepatic damage biomarkers

Statistical analysis of AST concentrations revealed a significant effect of time (F_(1, 21)_ = 79.41, p < 0.001, ƞp² = 0.79), yet no significant effects of intensity or intensity × time interaction were detected. AST increased after HIIT (+10.67%, p < 0.001) and MIIT (+7.76%, p < 0.001) without significant difference between protocols. Similarly to AST, only a significant effect of time was found (F_(1, 21)_ = 24.73, p < 0.001, ƞp² = 0.54) for ALT. ALT levels increased after both HIIT (+6.40%, p < 0.001) and MIIT (+4.80%, p < 0.01). The Friedman test revealed a significant effect for CK (test = 16.72, p < 0.001, Kendall’s W = 0.25). The Wilcoxon test showed an increase in CK levels after HIIT (+10.12%, p < 0.001) and MIIT (+8.57%, p < 0.001) without significant differences across sessions. There was only a significant effect of time (F_(1, 21)_ = 40.4, p < 0.001, ƞp² = 0.66) for LDH. The Bonferroni post-hoc test showed a significant increase of LDH after HIIT (+7.85%, p < 0.001) and MIIT (+9.67%, p < 0.001) without a significant difference between sessions (Table 4).

### Acute effects of HIIT and MIIT on BLa level

The Friedman test revealed a significant effect on BLa (test = 52.57, p < 0.001, Kendall’s W = 0.8). The Wilcoxon test showed an increase in BLa levels after both HIIT (+280.53%, p < 0.001) and MIIT (+208.93%, p < 0.001) with a greater increase following HIIT when compared to MIIT (p = 0.04) (Table 4).

### Acute effects of HIIT and MIIT on perceptual responses

**FIG. 3:**
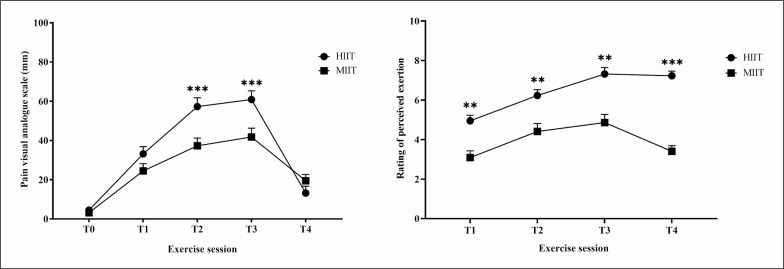
Changes in perceived pain and in rating of perceived exertion in response to acute HIIT and MIIT. Data are presented as mean ± SEM. Abbreviations: RPE, rating of percieved exertion; HIIT, high-intensity interval training; MIIT, moderate-intensity interval training; T1, after the first bout; T2, at the middle of the session; T3, after the last bout; T4, 3 min post-exercise. **, ***: significant difference between HIIT-MIIT at the same time point (p < 0.01, p < 0.001 respectively).

Pain scores differed between HIIT and MIIT (test = 152.07, p < 0.001, Kendall’s W = 0.77). Pain scores were higher in HIIT compared to MIIT at T2 (5.73 ± 0.45 versus 3.73 ± 0.40, p < 0.001) and at T3 (6.09 ± 0.44 versus 4.18 ± 0.45, p < 0.001) (Figure 3).

The Friedman test showed a significant main effect for RPE (test = 135.53, p < 0.001, Kendall’s W = 0.68). RPE revealed a greater increase with HIIT at T1 (4.95 ± 0.28 versus 3.09 ± 0.34, p < 0.01); T2 (6.23 ± 0.29 versus 4.41 ± 0.40, p < 0.01); T3 (7.23 ± 0.32 versus 4.86 ± 0.41, p < 0.01); and T4 (7.23 ± 0.24 versus 3.41 ± 0.28, p < 0.001) compared to MIIT (Figure 3).

## DISCUSSION

The present study aimed to compare the acute effects of two different interval exercise modes (HIIT and MIIT), matched for duration and total workload on immune, haematological, biochemical, and perceptual responses in sedentary adults with obesity. The main findings of this study were as follows: only HIIT induced a significant increase in inflammatory markers (CRP, WBC and neutrophils), both HIIT and MIIT increased markers of cellular damage (AST, ALT, CK and LDH) and greater metabolic (BLa) and perceptual (pain and RPE) responses were recorded following HIIT compared to MIIT in sedentary adults with obesity.

In the present study, the increase in CRP levels only following the HIIT session agrees with the findings of Fernandes et al. [19], who observed an increase in CRP following acute cycling exercise (65% VO_2_peak for 50 min) in overweight patients with coronary artery disease. It is well known that obesity is associated with a low-grade systemic inflammation characterized by increased levels of pro-inflammatory cytokines including IL-6 and IL-1β, which are the main CRP enhancers [20]. Admittedly, strenuous exercise induces an increase in circulating pro-inflammatory cytokines such as IL-1β and TNF-α as well as a potent increase in IL-6 [21–23]. However, Kaspar et al. [24] reported unchanged levels of CRP after one session of HIIT and MICT in healthy, normal-weight males. A possible explanation for these equivocal outcomes is that baseline CRP levels are already elevated in people with obesity [20] and that HIIT was strenuous enough to exacerbate the inflammation.

An increase in WBC was observed only after HIIT, indicating that this increase in response to interval exercise can be dependent on its intensity, which could be mediated by a greater increase of catecholamine levels and their interaction with beta-adrenergic receptors after sympathetic nervous system activation [25]. Likewise, Neves et al. [26] found an intensity dependence for WBC immediately after exercise when comparing low- versus high-intensity continuous exercise bouts, in healthy normal-weight men. In the present study, the leukocytosis in response to HIIT was basically due to neutrophilia. Similarly, Durrer et al. [27] reported a rapid increase of neutrophil number after a HIIT session in patients living with obesity and type 2 diabetes. It is known that acute exercise induces a rapid initial increase in circulating neutrophils possibly attributable to shear stress and catecholamines, followed by a delayed elevation through neutrophil release from the bone marrow likely mediated by cortisol [28–30]. Indeed, obesity is linked to a vegetative nervous system imbalance, characterized by high sympathetic nervous system activity and low parasympathetic nervous system activity [31], which could be aggravated following intense exercise. In fact, differences in acute immune system responses to HIIT and MIIT could be in part explained by a greater increase in the sympathetic nervous activity following HIIT compared to MIIT, since the magnitude of the sympathetic response to one exercise session depends on exercise intensity (i.e., the higher the exercise intensity, the greater the sympathetic activity) [32]. Increased sympathetic nervous system activity causes an increase in the secretion of catecholamines, which are highly implicated in the mobilization of leukocytes [25]. Another mechanism that could explain these findings is the increase of hypothalamic–pituitary–adrenal axis activity in response to one session of HIIT but not MIIT, which was demonstrated to be moderated by exercise intensity [33]. In fact, it has been reported that only a HIIT session induced an increase in plasma adrenocorticotropin levels when compared to one session of MIIT [33] or MICT [34]. The increase in adrenocorticotropin levels in response to pituitary gland stimulation by corticotropin-releasing hormone triggers the adrenal glands to raise cortisol levels, which is strongly implicated in leukocyte trafficking [25].

Inflammation following vigorous exercise has previously been linked to cell damage [35]. Exercise-induced muscle injury stimulates a variety of skeletal muscle cell types, including immune cells, to begin tissue repair and regeneration [36]. In the present study, an immediate elevation in all biomarkers of muscle damage (e.g. CK, LDH) after both interval exercises was reported. These findings are in line with those of Wiewelhove et al. [37] demonstrating that short-maximal and long-submaximal HIIT sessions induced an increase in CK levels in intermittent sports athletes. Dorneles et al. [9] observed an increase in CK levels immediately after one session of HIIT and MIIT, yet LDH increased only following HIIT in males with overweight/obesity. This disagreement regarding the increased level of LDH after MIIT in our study could be due to differences in the duration of work/rest intervals used in MIIT [38]. In fact, when the exercise load surpasses the muscle’s ability or when performing an unaccustomed exercise, both CK and LDH leak from the muscle cells into interstitial fluid and enter the bloodstream through the lymphatic system [35, 39]. Our findings showed a similar elevation of ALT and AST after both exercise modes. AST is found in the liver as well as in skeletal muscle and other tissues, while ALT is mainly present in the liver with smaller amounts in other tissues including skeletal muscle [40]. Thus, in the absence of liver diseases, and when confirmed with a simultaneous increase in levels of CK and LDH, AST and ALT should refer to muscle injury instead of liver injury [41]. One session of unaccustomed high-intensity exercise can induce disruption of the sarcolemma and liberate intramuscular protein including aminotransferases into the serum [42]. Interestingly, it has been reported that obesity could negatively affect markers of cell damage following intense exercise [43]. Previous studies reported that the group with a high body fat percentage [43] or BMI [10] had a greater increase in markers of cell damage after intense repetitive exercise (i.e., eccentric exercise) compared to their lean counterparts. In fact, it has been demonstrated that excessive fat accumulation increases fatty acyl chain saturation in the sarcolemma as well as phospholipid packing density, resulting in a rigid cell membrane that is more vulnerable to damage [44]. Moreover, obesity-related low-grade inflammation and oxidative stress facilitate the infiltration of phagocytes of injured muscle tissues, resulting in further cell disruption [45]. Another predictor of muscle damage is BLa [46]. In this study, a higher BLa level was observed after the HIIT session than after the MIIT session. According to the suggested model for HIIT categorization by Laursen and Buchheit [47], MIIT can be classified as aerobic since the post-exercise level of BLa was greater than 3 mmol/L and HIIT as moderately anaerobic since BLa was greater than 4 mmol/L; hence a higher BLa accumulation could be due to a greater draw on the anaerobic glycolytic system for energy production during HIIT. In addition, Warr-di Piero et al. [48] reported a higher BLa concentration after one HIIT session involving long interval durations (90 s/90 s and 130 s/130 s) compared to short intervals (10 s/10 s and 50 s/50 s) performed at 100% of maximal aerobic speed. These results do not support our findings regarding the effect of work interval duration on BLa as work bouts in HIIT were one-third shorter than MIIT but performed at a greater workload (i.e. 90%PPO versus 70%PPO), which suggests that not only the duration of work interval but also the work intensity could affect BLa concentration.

Regarding perceptual responses, our findings showed a greater RPE and pain in the HIIT session versus the MIIT session. In agreement with the findings of Wiewlhove et al. [37], pain scores were higher after all-out sprint IT than after long submaximal interval exercises. It has been previously reported that strenuous exercise inducing an elevation in muscle damage markers is associated with increased perceived pain and soreness after exercise cessation [49]. HIIT was perceived as “very difficult” and MIIT was perceived as “moderate” by inactive adults with obesity despite lower actual work (i.e. 8 min in HIIT versus 15 min in MIIT). Although Warr-di Piero et al. [48] demonstrated higher RPE during long versus short HIIT, it is worth mentioning that work duration was extended from one protocol to another while the work intensity was constant (100% of maximum aerobic speed), which could increase the RPE by participants. Our findings are in line with those of Wood et al. [50] reporting higher RPE in sprint IT versus HIIT, which may be due to a greater contribution of the higher intensity of IT in determining perceptual responses.

Nevertheless, the current study has some limitations. First, the measurements of biochemical markers were done only before and immediately after exercise. Second, the number of participants was relatively small. Third, we did not control for the effect of menstrual cycle phases on inflammatory responses, yet all sessions were not carried out during menses. Indeed, discrepancies between findings have been reported in previous studies conducted on eumenorrheic women, demonstrating no effect of menstrual cycle on CRP and TNF-α [51], while another study showed that levels of CRP vary across the menstrual cycle and reach the highest levels during menses [52]. Further studies are needed to increase the follow-up time after exercise and to study the chronic effect of MIIT and HIIT in inactive adults living with obesity.

## CONCLUSIONS

The present study demonstrates that one IT session induced an increase in biochemical and perceptual responses and these alterations depend on IT intensity, with a larger, acute increase after HIIT. The MIIT session induced fewer immune system perturbations and less muscle fatigue and was perceived as more tolerable compared to HIIT. Therefore, in sedentary people with obesity, MIIT could be used as a first step to favour body adaptations to a further HIIT programme.
